# Phage–Plant Interactions: A Way Forward toward Sustainable Agriculture

**DOI:** 10.3390/v15020329

**Published:** 2023-01-24

**Authors:** Temoor Ahmed, Bin Li

**Affiliations:** State Key Laboratory of Rice Biology, Ministry of Agriculture Key Laboratory of Molecular Biology of Crop Pathogens and Insects, Key Laboratory of Biology of Crop Pathogens and Insects of Zhejiang Province, Institute of Biotechnology, Zhejiang University, Hangzhou 310058, China

Agriculture is the most important sector as it provides food to the growing global population. Agricultural crops are severely affected by plant-bacterial pathogens, resulting in a significant reduction in plant growth and productivity [1]. Over the past few decades, chemical pesticides and antibiotics have been widely used for plant disease management; however, these options are environmentally damaging, expensive, lead to pathogen resistance, and pose severe threats to human health and food safety. Hence, there is an urgent need to develop novel techniques to control bacterial plant diseases and combat food insecurity. Bacteriophage virus (phage) therapy has recently attracted attention as a cost-effective and eco-friendly strategy to improve crop protection against bacterial infection, without affecting flora and fauna. Lytic phages can provide specific, nontoxic, and antibacterial action against targeted bacterial pathogens. In this special issue, active research efforts investigating the potential of bacteriophages as biocontrol agents against economically important bacterial phytopathogens are reported. 

This Special Issue contains four original research articles and two review articles that contribute to the knowledge of phage–plant interactions. Farooq et al. [1] reviewed numerous underlying mechanisms of phage–bacterial pathogen interactions, enabling the formulation of effective phage cocktail therapies against phytobacterial pathogens for plant disease management. They also highlighted the recent development and application of phage-mediated biocontrol strategies to combat resistant plant-bacterial pathogens. Zhang et al. [2] investigated the functions of lysis proteins, endolysin (LysAP) and holin (HolAP), in *Acidovorax oryzae* phage AP1, identifying a new binary lysis cassette. LysAP can be transported to the periplasm using its C-terminal TMD and Sec system. Moreover, the interaction of HolAP with LysAP, that acts as a possible pinholin, improves lysis efficiency. Overall, the authors demonstrated the phage–bacteria interaction mechanism, and provided novel insights in understanding phage life cycles and biocontrol of bacterial pathogens. 

In another study, Zhang et al. [3] revealed that a spontaneous frameshift leading to a premature stop mutation of a gene CDS2289 encoding a glycosyltransferase conferred *Xanthomonas oryzae* pv. *Oryzae* (Xoo) resistance to lytic phage X2 by altering lipopolysaccharide (LPS) production, bacterial motility, and surface morphology. This study showed the role of glycosyltransferase in interactions among phages, pathogenic bacteria, and plant hosts using phenotypic and genomic analysis. Retamales et al. [4] characterized the *Xanthomonas arboricola* pv. *juglandis* (Xaj) phages (*f29*-Xaj, *f20*-Xaj, and *f30*-Xaj) and evaluated their effectiveness against walnut blight disease under field conditions in Chile. Additionally, they suggested that the application of phage cocktails is a promising strategy to combat walnut blight disease. 

Liu et al. [5] identified three genes that are linked to phage sensitivity and host pathogenicity of the bacterial pathogen Xoo, which causes rice bacterial leaf blight disease. All three genes are involved in lipopolysaccharide production, which alters the surface chemical composition of the cell envelope. Luo et al. [6] summarized the current understanding of the isolation of *Pseudomonas syringae* pv. *actinidiae* (Psa) phages and their characterization, such as their morphology, lytic activity, lysis mechanism, host range, and genome characterization. In addition, they also described the application strategies of phages to control bacterial canker disease in kiwifruit, together with the potential challenges of phage therapy.

In summary, the article collection in this Special Issue provides valuable results and new views on the topic of phage–plant interactions. All authors also emphasized the need for further research and indicated future study directions. Overall, this Special Issue demonstrates that phage therapy serves as a non-toxic, sustainable, and highly efficient alternative for plant disease management.
